# A Case of Perforating Folliculitis in a Peritoneal Dialysis Patient

**DOI:** 10.5811/cpcem.1470

**Published:** 2024-01-09

**Authors:** Glenn Goodwin, Katherine L. O’Neil, Megan Dekok, Moshe Bengio, Philip O. Scumpia, Abhishek Roka, Alexander J. Scumpia

**Affiliations:** * HCA Florida Aventura Hospital, Department of Emergency Medicine, Aventura, Florida; † Rocky Vista University College of Osteopathic Medicine, Parker, Colorado; ‡ University of California Los Angeles David Geffen School of Medicine, Division of Dermatology/Dermatopathology, Los Angeles, California

**Keywords:** *case report*, *peritoneal dialysis*, *perforating folliculitis*

## Abstract

**Case Presentation:**

A 30-year-old male with a past medical history of hypertension and renal failure on peritoneal dialysis presented to the emergency department with a chief complaint of a rash on his anterior trunk for the prior three weeks. Dermatological examination revealed multiple, discrete folliculocentric, erythematous, and hyperpigmented papules, with scattered adjacent angulated erosions.

**Discussion:**

Perforating folliculitis is a rare and often difficult to diagnose skin condition classically seen in patients with chronic renal disease or underlying immunodeficiency.

Population Health Research Capsule What do we already know about this clinical entity?

*Perforating folliculitis is classically seen in patients with chronic renal disease or underlying immunodeficiency.*

What is the major impact of the image?

*This case highlights the importance of broadening the differential for undifferentiated rashes that present to the emergency department.*

How might this improve emergency medicine practice?

*Awareness of the association between the patient’s receiving peritoneal dialysis and the development of perforating folliculitis can expedite patient care.*

## CASE PRESENTATION

A 30-year-old male with a past medical history of hypertension and renal failure on peritoneal dialysis presented to the emergency department (ED) through triage with a chief complaint of a pruritic rash on his anterior trunk for the prior three weeks. The patient had complaints of multiple dark, erythematous, raised, pruritic lesions on the lower chest and anterior abdomen. He denied any new exposures, new medications, or recent travel. Symptomatic management was not initiated prior to presenting to the ED. The patient also denied associated systemic symptoms. Routine laboratory results were within normal limits. Dermatological examination revealed multiple, discrete folliculocentric, erythematous, and hyperpigmented papules with scattered adjacent angulated erosions (Image). The rash spared mucosal surfaces with no signs of contiguous spread onto the limbs, palms, or soles.

**Image.**
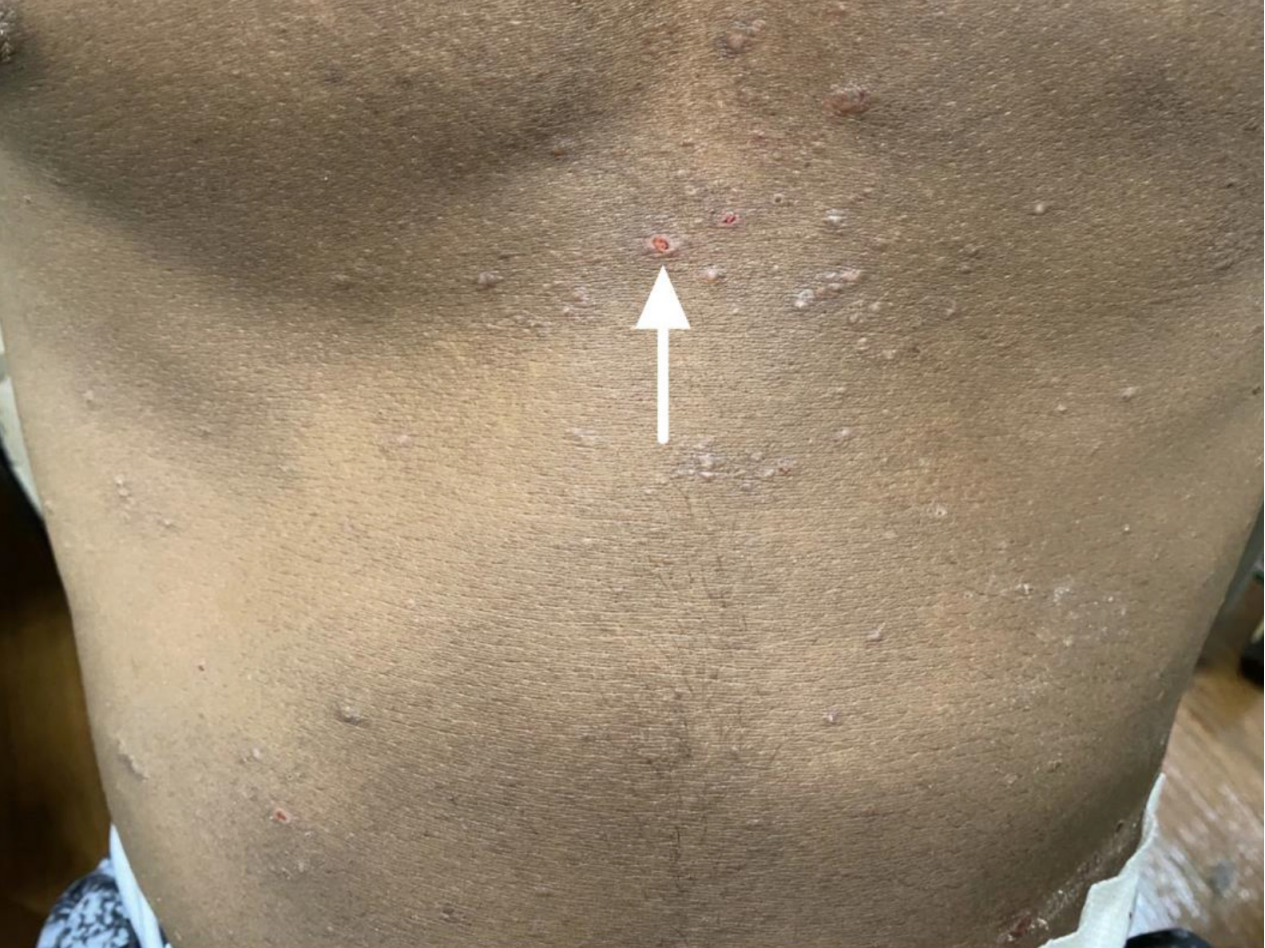
30-year-old male with multiple, discrete folliculocentric, erythematous, and hyperpigmented papules on the anterior chest consistent with perforating folliculitis (arrow).

## DISCUSSION

Perforating disorders are characterized by transepidermal extrusion of altered keratin or other dermal connective tissue products and include four main conditions; however, the secondary form of this collection of diseases is regarded as acquired perforating dermatosis (APD)^1^ ^,^ ^2^ The only way to differentiate among the four conditions is through histopathological assessment, but clinical diagnosis of APD is often sufficient in the setting of a phenotypical assessment of the lesions along with the patient having diabetes and/or chronic renal failure.^2^ Other common associated comorbidities include diabetes, vitamin A deficiency, and HIV.^2^ ^–^ ^4^

The diagnosis of perforating folliculitis can be challenging. Emergency physicians are trained to recognize well-known, life-threatening rashes; however, they must also be cognizant of more benign rashes. While not life-threatening, these rashes can be debilitating and cause severe patient discomfort, necessitating accurate diagnosis to administer proper care and management. Our patient was treated with topical 0.1% triamcinolone lotion and given outpatient dermatology follow-up upon discharge from the ED. Perforating folliculitis is often treated with systemic or topical corticosteroids, retinoids, and keratolytic agents such as urea or salicylic acid.^5^ The pruritic symptoms are often treated with emollients and oral antihistamines.^5^
